# Data comparing the kinetics of procollagen type I processing by bone morphogenetic protein 1 (BMP-1) with and without procollagen C-proteinase enhancer 1 (PCPE-1)

**DOI:** 10.1016/j.dib.2016.10.027

**Published:** 2016-11-03

**Authors:** Laura Moschcovich, Efrat Kessler

**Affiliations:** Maurice and Gabriela Goldschleger Eye Research Institute, Tel-Aviv University Sackler Faculty of Medicine, Sheba Medical Center, Tel-Hashomer 52621, Israel

**Keywords:** Collagen biology, Extracellular matrix, Proteolytic processing, Procollagen C-proteinase, Bone morphogenetic protein 1, Procollagen C-proteinase enhancer, Enzyme kinetics

## Abstract

This article provides kinetic constants for C-terminal processing of procollagen type I by bone morphogenetic protein 1 (BMP-1; the major procollagen C-proteinase), a reaction stimulated by the connective tissue glycoprotein procollagen C-proteinase enhancer 1 (PCPE-1). Reported are *K*_*m*_, *V*_*max*_, *K*_*cat*_ and *K*_*cat*_/*K*_*m*_ (catalytic coefficient) values for BMP-1 alone, BMP-1 with intact PCPE-1, BMP-1 with the CUB (Complement C1r/C1s, Uegf, BMP-1) domains fragment of PCPE-1 as well as its NTR (netrin-like) domain.

**Specifications Table**

**Table:** 

Subject area	Biochemistry, Biology
More specific subject area	Collagen maturation; Enzyme kinetics
Type of data	Figures, Table
How data was acquired	Measurements of the rate of release of the C-propeptide from procollagen type I by bone morphogenetic protein 1 (BMP-1) at different procollagen concentrations and calculation of kinetic constants using Lineweaver-Burk plots and in part also the Michaelis–Menten equation using Graphpad software.
Data format	Analyzed
Experimental factors	Chick embryo tendon procollagen type I was labeled biosynthetically with H^3^-tryptophan. The CUB (Complement C1r/C1s, Uegf, BMP-1) and netrin-like (NTR) domains fragments of PCPE-1 were generated by limited proteolysis with trypsin and isolated by heparin-Sepharose chromatography.
Experimental features	Proteolytic processing was quantified by measuring the amount of radioactivity in the procollagen C-propeptide released by BMP-1.
Data source location	Tel Aviv University, Tel Aviv, Israel
Data accessibility	Data is provided within this article

**Value of data**

• Researchers can use the data in designing in vitro assays of procollagen C-proteinase activity, in particular range of procollagen, BMP-1 and PCPE-1 concentrations.
• The data extends understanding of the mode of action of PCPE-1 and thus could suggest new research directions.
• The data rejects an early contradictory report on how PCPE-1 affects the kinetics of procollagen type I processing by BMP-1, thereby resolving a controversy in the field, also important as a guideline for future research.

## 1 Data

**Fig. 1:**
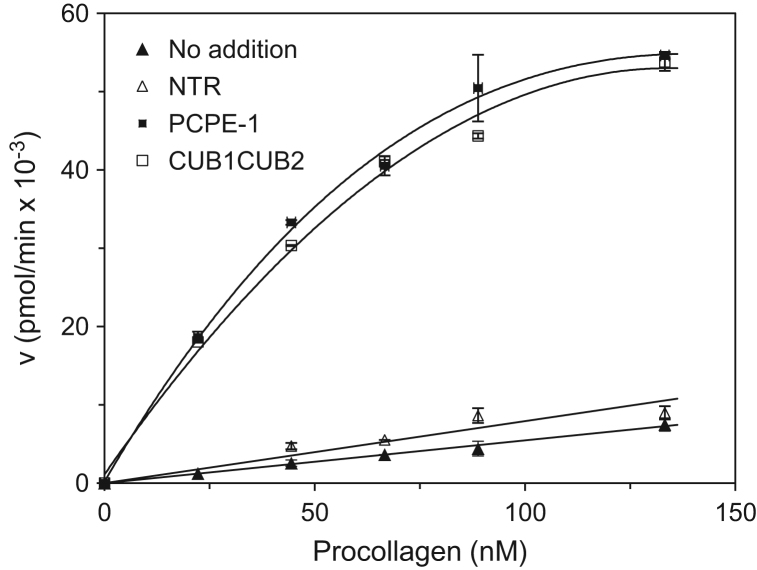
Initial rates of procollagen type I cleavage by BMP-1 as a function of procollagen concentration. Procollagen (0–133 nM) was incubated with BMP-1 alone (▲), BMP-1 plus intact PCPE-1 (■), BMP-1 plus the PCPE-1 CUB1CUB2 fragment (□), or BMP-1 plus the PCPE-1 NTR fragment (Δ). Each data point represents the average of two independent measurements except for BMP-1 alone, in which case, each point represents the average of six measurements. The error bars correspond to standard deviations (SDs).

**Fig. 2:**
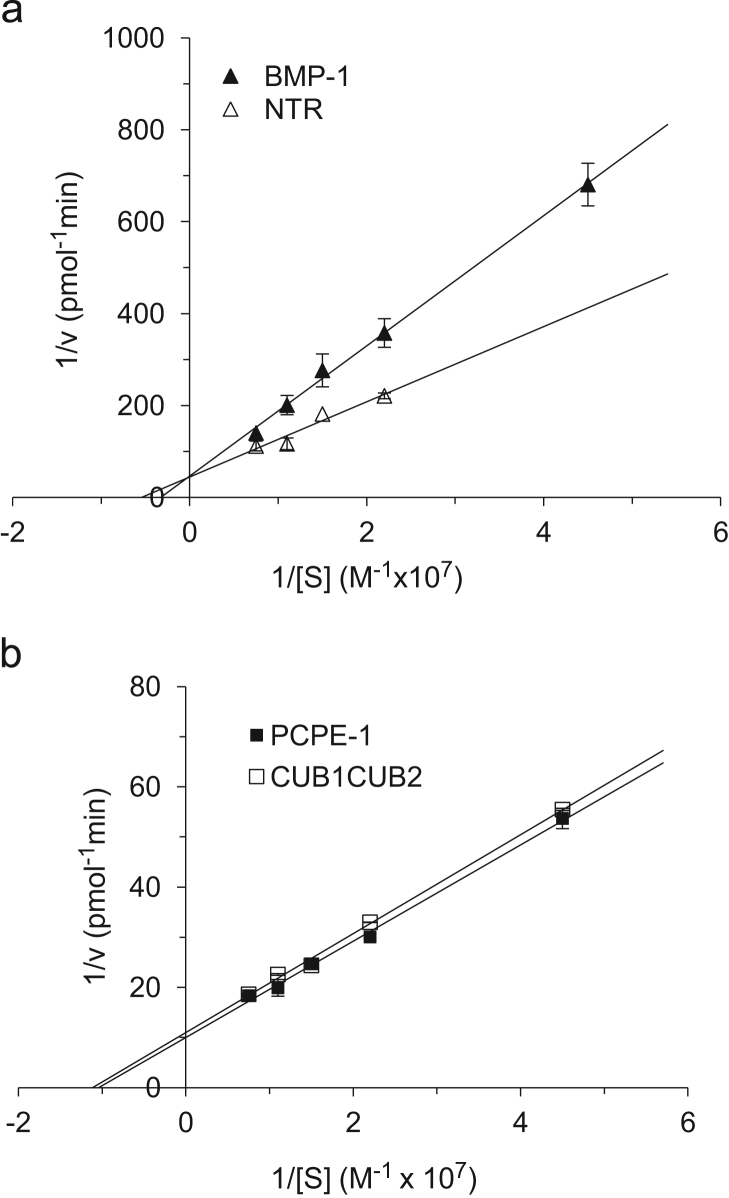
Lineweaver-Burk plots for BMP-1 in the absence or presence of the NTR domain (a) and in the presence of either intact PCPE-1 or its CUB domains fragment (b). The data were derived from the results shown in Fig. 1 except for BMP-1 alone, in which case, for consistency, the curve shows results of a representative experiment (out of 3 independent experiments, all of which yielded similar results). Thus, each point in all of the four curves represents an average of two measurements (*n*=2). The error bars correspond to SDs and are barely seen in panel b in which the curves show results obtained in the presence of the active forms of PCPE-1, conditions yielding high reaction rates and thus high accuracy (duplicates are essentially identical).

The data include a Figure depicting the initial rates of procollagen I cleavage by BMP-1 in the absence and presence of intact PCPE-1, its CUB domains fragment and its NTR domain as a function of procollagen concentration (Fig. 1). Also included are a Figure displaying the corresponding Lineweaver–Burk plots (Fig. 2) and a Table presenting the resulting *K_m_*, *V_max_, K_cat_* values and the related catalytic coefficients (*K*_*cat*_/*K*_*m*_) (Table 1).

## 2 Experimental design, materials and methods

### 2.1 Proteins

^3^H-tryptophan-labeled procollagen type I (specific activity 10.36×10^6^ cpm/mg) was prepared from chick embryo tendon fibroblasts [1]. The specific activity of the C-propeptide (4.7×10^7^ cpm/mg) was calculated assuming the molecular weight of the C-propeptide is 22% of that of intact procollagen. Recombinant human PCPE-1 was prepared as previously described [2]. The NTR and CUB1CUB2 domains of PCPE-1 were generated from it by limited proteolysis and isolated by chromatography on a heparin-Sepharose column [3]. Recombinant human BMP-1 was expressed in insect cells (baculovirus system) and purified as described [4].

### 2.2 Assay of procollagen C-proteinase activity and determination of kinetic constants

Procollagen C-proteinase activity of BMP-1 was determined at procollagen concentrations ranging from 22.2 to 133.2 nM (10–60 μg/ml) and a constant BMP-1 concentration of 0.14 nM estimated from relative band intensity after SDS-PAGE and silver staining. Due to the limited accuracy of such estimation the actual concentration of BMP-1 could be up to 50% higher or lower than the above value of 0.14 nM but this does not affect the ratios between kinetic constants with and without PCPE-1, the information we sought. Reaction conditions and measurements of the amount of radioactivity in the C-propeptide were as previously described [1], [5]. PCPE-1 and its fragments (when added to the reaction solutions) were used at 15 μg/ml (0.3, 0.5 and 0.75 μM for intact PCPE-1, the CUB1CUB2 fragment and the NTR fragment, respectively; molar ratios relatively to the highest procollagen concentration were 2.25, 3.7 and 5.6:1, respectively). This ensured that the amount of each of the PCPE-1 forms added would exceed saturation required to achieve maximal activity. The enzyme input was selected based on preliminary assays, ensuring that the reaction rates remained linear at all procollagen concentrations both in the absence and presence of PCPE-1. Reactions were initiated by enzyme addition and were conducted in duplicates both with and without BMP-1 to determine (and subtract) the background values at each procollagen concentration. The amount of radioactive C-propeptide released into the supernatants was calculated based on its specific activity. The kinetic constants *K_m_* and *V_max_* were derived from Lineweaver–Burk plots Fig. 2 and in the case of reactions in the presence of intact PCPE-1 and its CUB domains fragment, also based on the Michaelis-Menten equation achieved by fitting to the experimental data using Prism 5.0 (Graphpad software). This software did not allow calculations of kinetic constants for either BMP-1 alone or BMP-1 plus the NTR fragment, which required prohibitively high procollagen concentrations.

### 2.3 Statistical analysis

Data are presented as means±SD (standard deviation). Statistical significance was determined using two tail paired *t*-tests. Differences between groups were considered significant for *p* values <0.05.

## Figures and Tables

**Table 1:** Kinetic constants for procollagen type I processing by BMP-1 in the absence or presence of various PCPE-1 forms.

**Addition**	***K***_***m***_**(Mx10**^**−7**^**)**	***V***_***max***_**(M/hx10**^**−9**^**)**	***K***_***cat***_**(h**^**−1**^**)**	***Kcat/Km*****(h**^**−1**^**M**^**−1**^**×10**^**7**^**)**
None (*BMP-1 alone*)	3.9±1.2	7.9±2.3	56.0±15.7	14.4 (1.0)
				
PCPE-1	1.0±0.0	30.0±0.2	212.8±1.5	212.8 (14.8)
	***0.8±0.1***	***31.8***⁎	***227.0±15.2***	***283.8*** (19.7)
				
CUB1CUB2	0.9±0.0	27.0±0.0	191.5±0.5	212.8 (14.8)
	***0.8±0.1***	***31.7***⁎	***226.0±10.2***	***282.5*** (19.6)
NTR	1.9±0.1	6.6±0.9	46.8±6.0	24.6 ( 1.7)

Data are presented as mean ± standard error *n* = 6 for BMP-1 alone and 2 for each of the other conditions. The differences between the *K*_*m*_ and *V*_*max*_ values for BMP-1 alone and BMP-1 plus NTR are statistically insignificant (*p*=0.11 and 0.51, respectively) and so are the differences between the *K*_*m*_ values for BMP-1 plus PCPE-1 and BMP-1 plus its CUB domains fragment (*p*=0.095). The difference between the corresponding *V*_*max*_ values is statistically significant (*p*=0.004). Differences between *V*_*max*_ for BMP-1 alone and BMP-1 plus PCPE-1 or BMP-1 plus its CUB domains are statistically significant (*p*=0.00097 and 0.0014, respectively). *K_cat_* values were calculated assuming the enzyme concentration was 0.14 nM. This value however was estimated based on the relative band intensity after SDS-PAGE and silver staining with an estimated error of up to 50%. Since all of the assays were performed using identical aliquots of BMP-1, the comparison between the respective *K*_*cat*_ and *K*_*cat*_*/K*_*m*_ values is valid. Values in brackets represent the ratios between catalytic coefficients in the presence and absence of the various PCPE-1 forms. Standard errors of 0.0 are given for consistent data presentation. Authentic values for the *K_m_* s of BMP-1+PCPE-1 and BMP-1+CUB1CUB2 were 0.028 and 0.002, respectively; authentic standard error value for *V_max_* of BMP-1+CUB1CUB2 was 0.007. Values in ***Bold*** were determined by fitting the data to the Michaelis–Menten equation using Prism 5.0 (Graphpad software);

⁎ No standard error is provided by the software.
