# Supplementary material for: Data comparing the kinetics of procollagen type I processing by bone morphogenetic protein 1 (BMP-1) with and without procollagen C-proteinase enhancer 1 (PCPE-1)
Source: Data Brief. 2016 Nov 3;9:883–7. doi: 10.1016/j.dib.2016.10.027 (PMC5109235; doi:10.1016/j.dib.2016.10.027)
Supplement: Supplementary file 1 — Supplementary material [file mmc1.docx]

Conflict of interest declaration

Conflicts of interest: **None**
